# Therapeutics Potential of Cronassial in Experimental Autoimmune Encephalomyelitis: Insights Into Glycosphingolipids and Humoral Immunity

**DOI:** 10.1155/tswj/9108462

**Published:** 2025-07-18

**Authors:** Gayane Ghazaryan, Hasmik Zanginyan, Maria Ghazaryan, Laura Hovsepyan

**Affiliations:** Laboratory Cell Technologies, Institute of Molecular Biology NAS RA, Yerevan, Armenia

**Keywords:** APO-1/Fas, CICs, Cronassial, cytokines, EAE, gangliosides, glycosphingolipids

## Abstract

Currently, significant attention is being paid to the study of the mechanisms underlying the development of multiple sclerosis (MS), especially factors related to humoral immunity, apoptosis, and sphingolipid metabolism processes. These factors play a key role in neuroinflammation and neurodegeneration during both the acute and chronic stages of the disease. The aim of this study was to investigate the concentration of proinflammatory cytokines (IL-1*β*, IL-6, and TNF*α*) in plasma, homogenates of the brain and spinal cord, serum circulating immune complexes (CICs), the apoptosis marker APO-1/Fas, and the content of glycosphingolipids during experimental autoimmune encephalomyelitis (EAE) and its treatment. The therapeutic agent used in this study was Cronassial, which contains mono-, di-, and trisialylated gangliosides. Our results indicate the significant role of elevated proinflammatory cytokine levels in the pathogenesis of EAE, which initiate the activation of the sphingomyelin cycle and subsequently trigger apoptosis processes. During the study, we observed an increased concentration of APO-1/Fas. Administration of the ganglioside-containing drug in vivo led to the normalization of the levels of the studied factors, demonstrating a neuroprotective effect. According to our findings, this drug regulates the metabolism of glycosphingolipids and the humoral immune factors that were studied.

## 1. Introduction

Diseases characterized by the destruction of myelin present significant challenges in clinical medicine, with multiple sclerosis (MS) being the most common among them. EAE in rodents serves as an experimental model for human MS [1, 2].

The process of demyelination in MS involves several components that contribute to myelin damage and disruption of nerve fiber function. Among these components are proinflammatory cytokines such as IL-1*β*, which activates inflammatory processes and promotes the activation of microglia and astrocytes, potentially exacerbating myelin damage [3]. IL-6 plays an important role in inflammation and immune activation, influencing T-lymphocyte differentiation and antibody production. TNF*α*, a key cytokine, activates microglia and contributes to myelin sheath destruction through the activation of inflammatory processes and cell apoptosis [4].

CICs, composed of antibodies, antigens, and complement components, activate immune responses and inflammation. In MS, these complexes contribute to immune-mediated myelin damage by activating cellular mechanisms such as opsonization and phagocytosis, leading to the destruction of myelin sheaths [5].

Microglial cells are the primary cellular population responsible for immune responses in the central nervous system (CNS). In MS, microglia become activated in response to inflammation and damage, releasing proinflammatory cytokines that further contribute to myelin destruction. Oligodendrocytes, the cells responsible for myelin formation in the CNS, are damaged during inflammation and impaired regeneration, leading to progressive myelin loss and neurological deficits [6].

T-cells, especially Th1 and Th17 helper T-cells, play a central role in the pathogenesis of MS. These cells activate immune responses against myelin by releasing cytokines such as IFN-*γ* (interferon gamma) and IL-17, which stimulate inflammation and myelin sheath damage.

Glycosphingolipids (GSLs) are important components of cellular membranes, particularly myelin sheaths, and play a key role in cellular function and signaling pathways. In MS, the metabolism of GSLs is disrupted, contributing to pathological processes.

Myelin, the main component of white matter in the brain and spinal cord, contains high levels of lipids, particularly GSLs such as gangliosides. In MS, the balance of GSLs in myelin membranes is disrupted, leading to structural instability and damage. Alterations in the composition of these lipids may impair cellular membrane functions, reducing the stability of myelin.

In MS, changes in the activity of enzymes such as sphingomyelinase and gangliosidase, which regulate GSL metabolism, have been observed. For example, increased sphingomyelinase activity can lead to the accumulation of sphingosine, which plays a role in apoptosis and inflammation. This may exacerbate demyelination processes.

One of the consequences of disrupted GSL metabolism is the activation of apoptotic pathways in myelin-producing cells. For instance, the accumulation of ceramides in myelin sheaths can trigger oligodendrocyte cell death, promoting the progression of demyelination.

Gangliosides, such as GM1, play an essential role in maintaining the structure and function of myelin. Changes in the concentration of these molecules can disrupt intercellular interactions, affecting the protective properties of myelin and increasing the vulnerability of nerve cells to inflammation and damage [7–9].

Some studies suggest that restoring normal GSL metabolism or using ganglioside-containing drugs may exert neuroprotective effects, restoring myelin function and preventing myelin sheath degradation, which present a promising direction for MS therapy [10].

The objective of this study was to investigate the concentrations of proinflammatory cytokines (IL-1*β*, IL-6, and TNF*α*) in blood serum and brain/spinal cord homogenates, serum CICs, apoptosis processes (APO-1/Fas), and GSL metabolism in the brain/spinal cord during EAE, as well as the therapeutic effect of “Cronassial,” a ganglioside-containing drug.

## 2. Materials and Methods

### 2.1. EAE Induction and Experimental Groups

EAE was induced by immunization with 50 *μ*L of rat spinal cord emulsion (preisolated MBP according to the protocol) + commercial complete Freund's adjuvant (CFA) (SIGMA, F5881) in a ratio of 1:2) into the hind paw pads of rats [11].

The active ingredient in Cronassial (Fidia Research Laboratories, Italy) is a mixture of gangliosides: monosialotetrahexosylganglioside (GM1 21 ± 10%, Mr1545), disialotetrahexosylganglioside (GD1a ± 40% and GD1b 16 ± 10%, Mr1830), and trisialotetrahexosylganglioside (GT1b 19 ± 10%, Mr 2127). The product is a whitish powder, soluble in water but not in organic solvents, stable at room temperature for at least 5 years. The ganglioside mixture Cronassial does not exert toxic effects. Cronassial does not exhibit detectable toxicity [12].

The experiment used adult male rats weighing 200–220 g, provided by the vivarium of the Institute of Molecular Biology of the National Academy of Sciences of the Republic of Armenia. The rats were kept under controlled conditions according to Protocol No. 05072021/1; 07/05/2021, issued by the Committee of the Institute of Molecular Biology of the National Academy of Sciences of the Republic of Armenia for the care and maintenance of animals. Physiological and clinical parameters of the experimental animals were evaluated in the study [13] (Table 1).

### 2.2. Serum Measurement of Proinflammatory Cytokines, CD95, CICs, and Whole Brain GSLs

The concentrations of interleukins were determined using commercial enzyme-linked immunosorbent assay (ELISA) kits. Rat TNF-*α* (Cat.No. 438204) and Rat IL-6 (Cat.No. 437107) ELISA kits were obtained from BioLegend (San Diego). The Rat IL-1*β* ELISA kit (Cat.No.CSB-E08055r) was sourced from Cusabio (Wuhan, China), as was the rat factor–related apoptosis (FAS) ELISA Kit (#MBS451586).

The level of CICs was expressed in international units (IUs) [14].

The amount of cerebrosides was determined by the carbohydrate component with resorcinol, and sulfatides were determined by the sulfate group in reaction with azure. The amount of sphingosine was determined by a color reaction with methyl orange at a wavelength of 415 nm. [15, 16]

### 2.3. Statistical Analysis

Statistical analysis of the experimental data was performed using SPSS Statistics 18 (SPSS Inc., United States) [17]. The data were analyzed using the Student's *t*-test for independent samples. A significance level of *p* < 0.05 was applied. Differences between groups were considered statistically significant if the *p* value was below this threshold.

## 3. Results

### 3.1. Neutral Glycolipids Study

**Figure 1:**
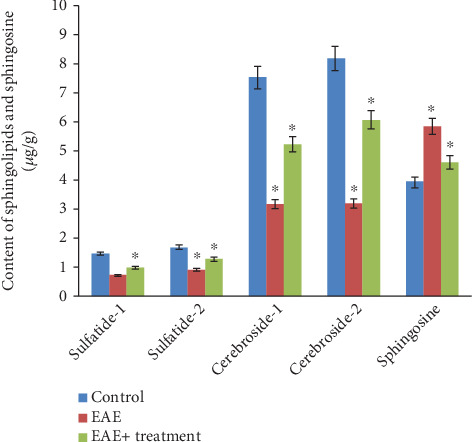
Content of sphingolipids and sphingosine in the homogenate of white matter of the brain under normal conditions, during EAE, and during therapy; ⁣^∗^*p* < 0.05 (*n* = 6).

**Figure 2:**
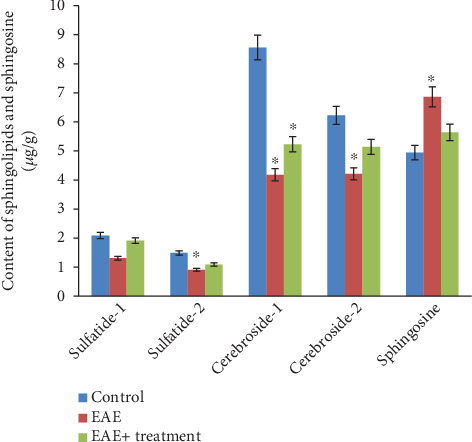
Content of sphingolipids and sphingosine in the homogenate of white matter of the spinal cord under normal conditions, during EAE, and during therapy; ⁣^∗^*p* < 0.05 (*n* = 6).

The analysis of neutral glycolipids revealed the presence of two cerebroside fractions and two sulfatide fractions in the white matter of the brain and spinal cord of experimental animals, indicating differences in their fatty acid composition (see Figures 1 and 2). Our research results showed a statistically significant (*p* < 0.05) decrease in both the total and fractional compositions of neutral glycolipids in the brain and spinal cord during EAE. In the brain, the total sphingolipid content was 19.17 *μ*g/g in the control group, which decreased to 10.61 *μ*g/g during EAE and subsequently increased to 13.37 *μ*g/g after the therapy. Sphingosine content increased during EAE compared to the control: 4.94 ± 0.21 in the control, 6.86 ± 0.22 during EAE, and decreased to 5.64 ± 0.44 after therapy. A similar trend was observed in the tissues of the spinal cord and brain: the total sphingolipid content was 18.91 *μ*g/g in the control, decreased to 8.01 *μ*g/g during EAE, and increased to 13.63 *μ*g/g after therapy. The sphingosine content was 3.94 ± 0.21 in the control, 5.86 ± 0.22 during EAE, and 4.64 ± 0.44 after therapy (*p* < 0.05).

### 3.2. Changes in Proinflammatory Cytokine and APO-1/Fas Levels in Serum and CNS Tissues of Experimental Animals With EAE: Effect of Cronassial Treatment

According to the results of our study, an increase in the levels of proinflammatory cytokines TNF*α*, IL-6, IL-1*β*, and APO-1/FAS was observed in the blood serum as well as in the homogenates of the brain and spinal cord of rats with EAE (Table 2).

The concentration of IL-6 (picograms per milliliter) in the blood serum was approximately twice as high in pathology (EAE in rats) compared to the control group. In the spinal cord, the concentration of IL-6 in the control group was 22.6 ± 4.2, whereas in pathology, it increased by 4.7-fold. In brain tissue, the concentration of IL-6 in the control group was 36.45 ± 6.8, and in pathology, it was significantly elevated (110.91 ± 22.5) compared to the control. The differences between groups were statistically significant (*p* < 0.05) (Table 1).

After treatment with the ganglioside-containing drug Cronassial, the concentration of IL-6 in the blood serum, as well as in the spinal and brain tissue homogenates, decreased compared to EAE pathology (*p* < 0.05).

The concentration of TNF*α* (picograms per milliliter) in the blood serum was almost one and a half times higher in pathology compared to the control, which corresponds to a 35.4% increase. In the spinal cord and brain tissue homogenates, an increase in TNF*α* concentration of 24.6% was also observed. After treatment with Cronassial, the concentration of TNF*α* in the blood serum and in the spinal and brain tissue homogenates significantly normalized.

The concentration of IL-1*β* (picograms per milliliter) in the blood serum in our studied pathology was nonspecific (control: 0.03 ± 0.0; EAE: 0.22 ± 0.05). However, significant changes were observed when studying this interleukin in the brain and spinal cord homogenates compared to the control: In the spinal cord, it was 1.41 ± 0.23 in the control and increased to 4.04 ± 0.81 in pathology; in brain tissue, it was 2.47 ± 0.58 in the control and increased to 6.15 ± 1.14 in pathology.

Following the administration of Cronassial in vivo, the obtained data indicate a regulatory process of proinflammatory interleukins (TNF*α*, IL-6, and IL-1*β*) concentrations in the blood serum and homogenates of the spinal cord and brain in experimental animals. However, their levels did not reach control values.

As shown by the results of our study, the expression level of APO-1/Fas was significantly higher during EAE compared to control animals. In the blood serum, the concentration of APO-1/Fas in the control was 0.05 ± 0.01 pg/mL, in EAE it was 0.47 ± 0.05, and the decrease during therapy was 14.9%. In the homogenate of the white matter of the brain and spinal cord, in the control, it was 0.33 ± 0.08; in EAE, it was 0.63 ± 0.1, and during therapy, it was 0.47 ± 0.11, which corresponds to a 25.4% decrease (*p* < 0.05).

### 3.3. CICs in EAE: Impact of Pathology and Treatment

**Figure 3:**
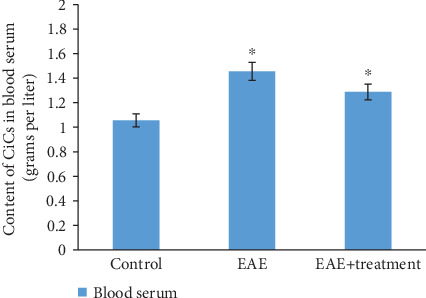
The content of CICs in blood serum under normal conditions, during EAE, and during treatment (grams per liter). ⁣^∗^*p* < 0.05 (*n* = 6).

As a result of the studies conducted on animals with modeled EAE, on the 21st day of pathology development, an increase in CIC concentration in blood serum is observed, approximately 1.5 times higher compared to the control: in the control group 1.30 ± 0.01, during the pathology 1.82 ± 0.09. With treatment, it was 1.61 ± 0.08 (Figure 3).

## 4. Discussion

In MS, inflammation in the CNS plays a key role in the pathogenesis of the disease. One of the important elements of this inflammation is cytokines—molecules that regulate the immune response and inflammation. Cytokines involved in the inflammatory process in MS can be either proinflammatory or anti-inflammatory [18]. Let us consider the main proinflammatory cytokines actively participating in the pathogenesis of MS:

TNF-*α* is one of the most potent proinflammatory cytokines, playing a central role in immune activation in MS. TNF-*α* promotes the activation of microglia and other immune cells, increases the permeability of the blood-brain barrier, and activates cellular cascades that lead to inflammation and myelin damage [19].

IL-1*β* also plays an important role in inflammation in MS. It stimulates the production of other cytokines, activates neurophages and microglia, increases barrier permeability, and promotes myelin degradation [20].

IL-6 is a key mediator of inflammation, contributing to T-lymphocyte activation and increasing antibody production. It also participates in the activation of immune system cells and can contribute to disease progression [21, 22].

Our study results confirm the decisive role of proinflammatory cytokines, such as TNF-*α*, IL-6, and IL-1*β*, in the pathogenesis of EAE. These cytokines activate inflammatory processes, leading to the activation of sphingomyelin cycles, which ultimately results in the apoptosis of CNS cells and subsequent neurodegeneration. Our findings show a significant increase in the levels of these cytokines in plasma and homogenates of the brain and spinal cord of EAE animals, which is consistent with the literature confirming their role in inflammation and myelin damage [4, 10].

APO-1/FAS is a molecule playing a key role in apoptosis or programmed cell death. FAS is a receptor activated when it binds to its ligand (FASL). This process regulates the cell life cycle, especially in the context of the immune system, where apoptosis helps maintain homeostasis and prevent autoimmune reactions. APO-1/FAS is particularly important for activating apoptosis in T-lymphocytes, which helps control the immune response and prevents excessive immune system activation. Disruption in the regulation of this mechanism can lead to various diseases, including autoimmune disorders such as MS. In MS, activated T-cells attack the myelin sheath, leading to damage to nerve fibers and impaired nerve impulse transmission.

The function of APO-1/FAS in MS involves the regulation of T-lymphocyte cell death, either preventing or enhancing their activity. If the apoptotic process is disrupted, it may lead to excessive activation of autoimmune cells and chronic inflammation, which underlies the pathogenesis of MS.

Studies show that the activity of APO-1/FAS and its ligand FASL can affect the course of MS. For example, increased expression of FAS can contribute to enhanced T-cell death, which may suppress inflammation and the immune response. Conversely, decreased activity of APO-1/FAS or mutations in this gene may lead to ineffective apoptosis activation, potentially promoting chronic inflammation and myelin damage [23].

Our study also revealed an increase in the level of APO-1/Fas, an apoptosis marker, which further confirms the involvement of apoptosis in the pathogenesis of EAE. According to the literature, the activation of the Fas receptor and its interaction with the Fas ligand initiates an apoptotic cascade leading to cell death, including oligodendrocytes, which are responsible for myelin formation [24]. The increase in APO-1/Fas levels in our study also supports the hypothesis that apoptosis contributes to myelin destruction in EAE. Disruptions in this mechanism can lead to excessive inflammation and myelin damage. Studying APO-1/FAS and its interactions with other immune system molecules can help develop new therapeutic strategies aimed at correcting the immune response in MS. However, further research is needed for a more precise understanding of this receptor's role in disease development and the development of effective treatments.

CICs are antigen-antibody complexes that can be detected in blood plasma. They form as a result of the interaction of antibodies with exogenous or endogenous antigens. Such complexes may be functional in response to infections or autoimmune reactions, where antibodies target the body's own tissues. CICs can be temporarily circulating or have a long-term presence in the bloodstream.

In MS, an autoimmune disease where the immune system attacks the myelin sheaths of neurons, CICs play a key role in pathogenesis. They can contribute to chronic inflammation, myelin damage, and CNS dysfunction [25].

CICs can directly promote myelin degradation through the following mechanisms:

- Complement activation: The production of complement components in response to immune complexes leads to the formation of anaphylatoxins such as C3a and C5a, which increase vascular permeability and damage myelin sheaths. This also activates myelin phagocytosis.
- T-lymphocyte recruitment: During inflammation caused by CICs, T-lymphocytes are activated, which, in turn, activate macrophages and other cells that damage myelin. These cells can also secrete proinflammatory cytokines (e.g., TNF-*α*, IL-1, and IL-6), further enhancing inflammation and neuronal damage [26].

An increase in CIC levels in the blood of EAE animals in our study is also consistent with previous research indicating their role in activating inflammation and myelin damage. Elevated CIC levels contribute to the activation of immune mechanisms such as opsonization and phagocytosis, leading to the destruction of myelin sheaths [5, 27].

In our study, we also observed an increase in the concentration of CIC in the blood of EAE animals, which was reduced after treatment with the drug Cronassial. This indicates its ability to reduce the immune response associated with CICs. CICs can serve as an important diagnostic and prognostic marker in MS. Studies show that elevated CIC levels may correlate with disease activity, disability progression, and relapse frequency. The level of CICs can be used to assess inflammatory activity and monitor treatment effectiveness.

On the other hand, excessive activity of immune complexes may also indicate a more aggressive form of MS, which requires special treatment approaches. In recent years, therapeutic approaches targeting CICs in MS have been actively studied. They can serve as both a marker of disease activity and a target for therapeutic interventions.

Treatment with ganglioside-containing drugs such as Cronassial led to the normalization of proinflammatory cytokine, APO-1/Fas, and CICs levels, indicating the neuroprotective properties of this drug. Specifically, the reduction in TNF-*α*, IL-6, and IL-1*β* levels in the blood and CNS tissues after administration of the drug confirms its ability to modulate inflammatory responses. This is consistent with other studies showing that gangliosides can regulate immune responses by suppressing Th1 cell activation and reducing inflammation [28, 29].

When comparing our results with the literature, several key aspects emerge. In particular, numerous studies confirm the role of GSL metabolism disturbances, such as gangliosides, in the development of inflammation and neurodegeneration in EAE [30–33]. Our results show that treatment with Cronassial normalizes the levels of GSLs such as cerebrosides and sulfatides in the brain and spinal cord tissues, indicating the restoration of structural integrity of cell membranes [34, 35]. The effect of Cronassial in reducing sphingosine levels, a product of GSL hydrolysis, also confirms the results of other studies showing that gangliosides can normalize GSL metabolism and reduce inflammation associated with elevated sphingomyelinase activity [36–38].

The mechanisms through which Cronassial modulates proinflammatory cytokine and CICs concentrations may be related to its ability to affect sphingomyelinase activity, which plays a key role in the sphingomyelin cycle and the activation of inflammatory processes. Sphingomyelinase, by activating the cycle, promotes the formation of sphingosine, which can initiate apoptosis in cells. The administration of Cronassial, containing gangliosides, may normalize sphingomyelinase activity, thereby reducing inflammation and apoptosis in CNS cells. This also explains its neuroprotective effect, which manifests in a reduction in APO-1/Fas levels and normalization of GSL metabolism.

Thus, our data, consistent with the results of other studies, confirm that Cronassial has neuroprotective properties by normalizing proinflammatory cytokine, CICs, and apoptosis marker concentrations, as well as restoring GSL metabolism. This makes it a promising therapeutic agent for treating diseases associated with neuroinflammation, such as MS.

## 5. Conclusion

Our study highlights the important role of proinflammatory cytokines in the pathogenesis of EAE, which activate the sphingomyelin cycle and lead to elevated ceramide and sphingosine levels, thereby initiating apoptosis processes. Administration of the Cronassial drug, containing gangliosides, normalizes the levels of these factors, demonstrating a neuroprotective effect. These results are consistent with existing literature and form the basis for further research into the use of ganglioside-containing drugs in the treatment of neuroinflammatory diseases. Cronassial appears to exert neuroprotective effects in EAE by modulating the sphingomyelin cycle influenced by proinflammatory cytokines and inhibiting apoptotic pathways.

## Figures and Tables

**Table 1:** Physiological indicators and symptoms: body weight changes in control, EAE-induced, and Cronassial-treated rats.

**Physiological indicators (symptoms)**	**Experimental groups**		
	**Control (** **n** = 6**)**	**EAE (** **n** = 20**) EAE was induced by immunization with 50 *μ*L of rat spinal cord emulsion (preisolated MBP according to the protocol) + commercial complete Freund's adjuvant (CFA) (SIGMA, F5881) in a ratio of 1:2) into the hind paw pads of rats**	**EAE+ Cronassial (** **n** = 6**) on the 22nd day with the development of characteristic signs of the disease. Ganglioside-containing drug Cronassial was administered to animals per os at the rate of 20 mg of the drug per kg of body weight (20 mg/kg)**
Body weight (g)	210 ± 6.58	170 ± 9.38 ⁣^∗^*p* = 0.027888 ^#^*p* < 0.05	191 ± 4.58; ⁣^∗^*p* = 0.026557; ^#^*p* < 0.05; ⁣^∗∗^*p* = 0.302920

⁣^∗^Compared to the control group.

⁣^∗∗^Compared to the pathology group.

^#^The differences between groups were statistically significant *p* < 0.05.

**Table 2:** The concentration of TNF*α*, IL-6, IL-1*β*, and APO-1/FAS (picograms per milliliter) in blood serum and in the homogenates of the spinal cord and brain of experimental animals in the control, in EAE, and during treatment; ⁣^∗^*p* ≤ 0.05 (*n* = 6).

**Absorbance (450 nm)**	**Control**			**EAE**			**EAE + treatment**		
	**Blood serum**	**Brain tissue**	**Spinal cord**	**Blood serum**	**Brain tissue**	**Spinal cord**	**Blood serum**	**Brain tissue**	**Spinal cord**
IL-6 (pg/mL)	24 ± 4.5	36.45 ± 6.8	22.6 ± 4.2	45.6 ± 8.5^∗^ *p* = 0.05	120.9 ± 22.5^∗^ *p* = 0.006	116.9 ± 21.8^∗^ *p* = 0.022	37.6 ± 7	83.61 ± 15.68^∗^ *p* = 0.02	83.7 ± 15.6^∗^ *p* = 0.043
IL-1*β* (pg/mL)	0.03 ± 0.01	2.47 ± 0.58	1.41 ± 0.23	0.22 ± 0.05^∗^ *p* = 0.004	6.15 ± 1.14^∗^ *p* = 0.0183	4.04 ± 0.81^∗^ *p* = 0.0122	0.06 ± 0.01^∗^ *p* = 0.012	3.52 ± 0.84	1.96 ± 0.21
TNF-*α* (pg/mL)	181.25 ± 33	124.4 ± 22.8	152.7 ± 27.8	245.36 ± 44.5	154.9 ± 28.3	185.15 ± 33.8	190.2 ± 34.7	130.75 ± 23.9	172.2 ± 31.4
APO-1/FAS (pg/mL)	0.05 ± 0.01	0.33 ± 0.08	0.28 ± 0.05	0.47 ± 0.05	0.63 ± 0.10^∗^ *p* = 0.0383	0.863 ± 0.05^∗^ *p* = 0.00002	0.40 ± 0.01	0.47 ± 0.11	0.66 ± 0.06^∗^ *p* = 0.00088

⁣^∗^The differences between groups were statistically significant *p* < 0.05.

## Data Availability

The data that supports the findings of this study are available in the supporting information of this article.

## Nomenclature

EAE: experimental autoimmune encephalomyelitis
MS: multiple sclerosis
CAF: complete Freund's adjuvant
CICs: circulating immune complexes
CNS: central nervous system
GZ: gangliosides
